# Supplementary material for: The Systems Biology Research Tool: evolvable open-source software
Source: BMC Syst Biol. 2008 Jun 29;2:55. doi: 10.1186/1752-0509-2-55 (PMC2446383; doi:10.1186/1752-0509-2-55)
Supplement: Additional file 1 — SBRT Archive. An archive of the current version of the Systems Biology Research Tool. [file 1752-0509-2-55-S1.zip › sbrt-1.4.0/doc/developers_guide/api/sbrt/kernel/io/ConflictingInputException.html]

ConflictingInputException


|  |  |  |
| --- | --- | --- |
| **Overview** \| **Package** \| **Class** \| **Use** \| **Tree** \| **Deprecated** \| **Index** \| **Help** | |  |
| **PREV CLASS**   **NEXT CLASS** | **FRAMES**    **NO FRAMES**     **All Classes** |  |
| SUMMARY: NESTED \| FIELD \| CONSTR \| METHOD | DETAIL: FIELD \| CONSTR \| METHOD |  |


---


## sbrt.kernel.io Class ConflictingInputException

```
java.lang.Object
  java.lang.Throwable
      java.lang.Exception
          java.lang.RuntimeException
              sbrt.kernel.io.ConflictingInputException
```

**All Implemented Interfaces:**: java.io.Serializable

---

``` public class ConflictingInputException extends java.lang.RuntimeException ```

This class is used to indicate conflicting inputs.

**Author:**
:   This class was written and documented by Jeremiah Wright
    while in the Wagner lab.

**See Also:**: Serialized Form

---

**Constructor Summary**

`ConflictingInputException(java.util.List<?> conflicts)`
  
          Constructs a new conflicting input exception.


| **Method Summary** | |
| --- | --- |
| `UnmodifiableList<?>` | `getConflicts()`             Returns the list of conflicting inputs. |

**Methods inherited from class java.lang.Throwable**

`fillInStackTrace, getCause, getLocalizedMessage, getMessage, getStackTrace, initCause, printStackTrace, printStackTrace, printStackTrace, setStackTrace, toString`

**Methods inherited from class java.lang.Object**

`clone, equals, finalize, getClass, hashCode, notify, notifyAll, wait, wait, wait`

**Constructor Detail**

### ConflictingInputException

```
public ConflictingInputException(java.util.List<?> conflicts)
```

:   Constructs a new conflicting input exception.

    **Parameters:**: `conflicts` - a list of conflicting inputs.


**Method Detail**

### getConflicts

```
public UnmodifiableList<?> getConflicts()
```

:   Returns the list of conflicting inputs.

    :   **Returns:**: the list of conflicting inputs.


---


|  |  |  |
| --- | --- | --- |
| **Overview** \| **Package** \| **Class** \| **Use** \| **Tree** \| **Deprecated** \| **Index** \| **Help** | |  |
| **PREV CLASS**   **NEXT CLASS** | **FRAMES**    **NO FRAMES**     **All Classes** |  |
| SUMMARY: NESTED \| FIELD \| CONSTR \| METHOD | DETAIL: FIELD \| CONSTR \| METHOD |  |


---
